# Prevalence and associated factors of caesarean section in Addis Ababa hospitals, Ethiopia

**DOI:** 10.11604/pamj.2019.34.136.16264

**Published:** 2019-11-07

**Authors:** Hiwot Tsegaye, Birehanu Desalegne, Biresaw Wassihun, Agegnehu Bante, kassahun fikadu, Megbaru Debalkie, Tomas Yeheyis

**Affiliations:** 1 Department of Nursing and Midwifery, College of Health Sciences, Addis Ababa University, Addis Ababa, Ethiopia; 2 Colleges of Medicine and Health Sciences, Arba Minch University, Arba Minch, Ethiopia

**Keywords:** Caesarean section, prevalence, Addis Ababa, Ethiopia

## Abstract

**Introduction:**

Caesarean section refers to the operation of delivering a baby through incisions made in the mother's abdominal wall and uterus. A caesarean section is medically indicated when a significant risk of adverse outcome for mother or baby is present. The objective of this study was to assess the prevalence and associated factors of caesarean section in Addis Ababa Hospitals, Ethiopia.

**Methods:**

Institutional based cross-sectional study design was employed on 298 women from between April and May 2017. Study subjects were selected using systematic random sampling by considering the number of delivery. A structured questionnaire was used to collect the data. The data were coded and entered into Epi data version 3.1 and the analysis was carried out in a statistical package for social science versions 22. Descriptive statistics for each variable and binary logistic regression analysis with 95% CI was carried out.

**Results:**

A total of 298 mothers were participated in the study with a response rate of 100%. The overall prevalence of caesarean section in this study was 38.3%. The multivariable analysis indicated that mother who had collage and above [AOR = 3.46 (95%CI; 1.2, 10.76)], giving birth in private health facility [AOR = 1.48 (95%CI; 1.84, 2.59)], and having risk factors [AOR = 2.86 (95%CI; 1.96, 3.42)], were some of the factors associated with caesarean section.

**Conclusion:**

The finding of this study showed that the prevalence of caesarean section was higher in women who gave birth in private health facility, mothers having risk factors, and mothers having educational status of diploma and above. Therefore, identifying risky group during antenatal care follow-up and restraining numbers of caesarean section in private health facilities is essential steps to reduce high prevalence of caesarean section.

## Introduction

Caesarean section is a surgical technique in which openings are made through a woman's abdomen and uterus to deliver her baby. Caesarean section (C-section) may be indispensable when vaginal delivery might pose a danger to the mother or baby [1] caesarean section is one of the most significant lifesaving procedures that played a key role to decline maternal and perinatal morbidity and mortality rate. Caesarean section rates are high and continue to rise in industrialized countries, however, the impact of guidelines and recommendations in curbing their growth has been limited [2]. In the world now 1 in 5 women give birth by caesarean section. The average global rate of caesarean section (CS) is 18.6%, ranging from 6.0% to 27.2% in the least and more developed regions, respectively [3]. In 1985, representatives of a study group convened by the World Health Organization wrote, “there is no justification for any region to have caesarean section rates higher than 10-15% [4]. Despite this recommendation, evidences suggest that the rates of CS are high in developing countries and are increasing, with wide variation between countries and between regions of the same country [5]”. In Ethiopia, overall institutional rate was 18%, which varied between 46% in the private sector and 15% in the public sector [6]. In Addis Ababa the rate of Caesarean section was increasing from 16%-22.9% as Ethiopian demographic health survey (EDHS) 2005-2014 report. It signifies the possibility of over-utilization of the service in the city [6-8]. In many developed countries, caesarean sections are increased and attention has focused on strategies to reduce its use due to the concern that higher caesarean section rates do not confer additional health gain but may increase maternal risk, have implications for future pregnancies and have resource implications for health service [9-11]. Caesarean sections conducted without clinical need can have adverse consequences for mothers and children. It increased the risk of adverse psychological sequelae, and problems in subsequent pregnancy, including uterine scar rupture greater risk of stillbirth and neonatal morbidity compared with vaginal delivery [12,13]. In low resources countries, like Ethiopia, on the other hand, lack of availability of, or access to, maternal health services and the corresponding underuse of caesarean sections (CS) are part of a web of factors predisposing to high maternal and perinatal morbidity and mortality [14]. Rationally, in such a background, there is concern that apparently inexorably rising rates of caesarean delivery have the potential to divert human and financial resources [15]. Currently there is much debate as to whether this surgical procedure should be performed for women without clear clinically acceptable indications [9,16,17]. American College of Obstetricians and Gynecologists, and the Society for Maternal Fetal Medicine, called for policy change to safely lower the rate of primary caesarean delivery [18]. In different literature, high caesarean section rates were found especially in the private sector [19]. Most previous studies were done on prevalence of caesarean section and focus only public health facility but there is inadequate research on factors associated with increase rate of caesarean section in both private and public health facility. Therefore it is important to understand the factors that drive the high caesarean section rates in order to put in place of interventions to reduce the rate. So the aim of this study was to assess prevalence and factors associated with caesarean section in Addis Ababa hospitals, Ethiopia.

## Methods

**Study area period:** the study was conducted in Addis Ababa, the capital city of Ethiopia and it is located in the heart land of the country with a total area of 527 km^2^. This region has an estimated density 5,535.8 people per square kilometer. Based on 2007 figure from Central Statistics Agency of Ethiopia, Addis Ababa has an estimated total population of 3.2 million projected for the year 2014 .The city has ten sub city and 116 woredas. There are 51 hospitals of which 6 are owned by Addis Ababa City Administration Health Bureau, 4 by Federal Ministry of Health, 1 by Addis Ababa University, 3 by the Nongovernmental organization, 3 by Defense Force and Police and 34 by private owners. Out of this 10 of them are MCH hospitals. There are about 84 health centers and around 700 private clinics out of this 75 are higher Clinics. This study was carried out among the mothers who gave birth in the selected hospital (three government hospitals, three private MCH hospitals and one nongovernment MCH hospital. The study was conducted from April 3 to May 30, 2017.

**Study design:** institution based cross sectional study was conducted.

**Source population:** all mothers who gave birth in Addis Ababa hospitals, Addis Ababa, Ethiopia

**Study population:** mothers who gave birth in selected Addis Ababa hospitals in study period which fulfill the inclusion criteria.

**Sample Size Determination:** the sample size was determined by using single population proportion formula by taken the previous known magnitude of CS which was 22.9% from EDHS 2014 and adding non-response rate of 10% and considering the assumption of a 95% confidence level, a 5% margin of error the required sample size was 298 mothers.

**Sampling procedure:** seven Health Institutions were randomly selected. Three government hospitals, three from private MCH hospitals and one from nongovernment MCH hospital. The allocation of the sample to health facilities was made proportionally based on the average number of clients who received childbirth services at each facility in the month preceding the data collection period. Individual study subject at each health facility was selected by systematic random sampling during data collection period until the required sample size at each health facility was obtained. The sampling interval k=3 was calculated by dividing the source population to the total sample size and this interval was used in all health facility to select study subjects. The first client was selected by simple random sampling among the first three maternity care users in the sampling frame.

### Operational definition

**Prevalence of caesarean section**: is the proportion of caesarean sections performed in a hospital to the total number of live births in a study area.

**Data collection tool:** data was collected using structured Amharic interviewer administered questionnaire and questionnaire checklist by review client chart. The questionnaire and checklist were adapted through reviewing of different kinds of literature and previous similar studies. The questionnaire was developed in English and then translated in to Amharic and back to English then review was made for consistency of translation of the language. The tool consists of two sections the first section was used to assess socio-demographic characteristics of the respondents, and the second section was used to assess obstetric characteristics of respondents.

**Data collection procedure and quality control:** data were collected by face to face interview using structured questionnaires and questionnaire checklist by review client chart. Seven diploma holder female midwives were recruited to collect the data, and three BSC holders nurse as supervisors from another area outside of study site. Before data collection data collectors and supervisors were trained on the objective, benefit of the study, individual's right, informed consent and techniques of the interview for one day. Before starting the actual data collection to assure the data quality high emphasis was given to designing data collection instrument, first the questionnaire was pre-tested on 30 (10%) of sample outside the study area. After pre-testing further adjustments to the data collection tool was made to improve clarity, understandability, and simplicity of the messages. All of the questionnaires were checked for completeness and accuracy before, during and after the period of data collection. Throughout the course of the data collection, interviewers were supervised, regular meetings were held between the data collectors and the principal investigator together in which problematic issues arising from interviews during the data collection and mistakes found during editing was discussed. The collected data was again reviewed and checked for completeness before data entry. Data entry format template was prepared and programmed by the principal investigator.

**Data analysis and interpretation:** first the collected data were checked visually for completeness and any incomplete or misfiled questions then the data was cleaned and entered into Epi Data version 3.1 software then it was exported to statistical package for social sciences (SPSS) version 22.0 software for analysis. Descriptive statistics were done and presented using tables and figures. Initially, bivariate logistic regression was carried out to see the association of each of the independent variables with the outcome variable. Thereafter, the multivariable logistic regression method was used. P-value of <0.05 and 95% confidence level was used as to declared statistical significance.

## Results

### Socio-demographic characteristics of respondent

From the total of 298 sampled mothers all were enrolled in the study giving a response rate of 100%. Mean age of the respondents was 27 years (SD± 4.72) with a minimum and maximum age of 16 and 48 respectively. The majority of the respondents, 195(65.4%) were within 20-29 years age group. The majority of participants 286 (96.0%) were married. The educational status of respondent showed that 107(35.9%) were college diploma and above. Concerning the occupation of the respondents 163 (54.7%) were housewives. The average monthly family income of respondents was 6786.00 birr (Table 1).

**Table 1:** socio-demographic characteristics of respondents who gave birth in public and private hospitals, Addis Ababa Ethiopia, 2017(n= 298)

Variable	Frequency	Percentage (%)
**Age in years**		
15-19	4	1.4
20-24	67	22.5
24-29	128	43.0
30-34	66	22.1
35-39	33	11.1
**Marital status**		
Married	286	96.0
Single	5	1.7
Separate	1	0.3
Divorce	5	1.7
Widowed	1	0.3
**Educational status**		
Illiterate	24	8.1
Primary(1-8)	81	27.2
Secondary(9-12)	86	28.9
College & above	107	35.9
**Occupation**		
Housewife	163	54.7
Government employee	53	17.8
NGO	6	2.0
Merchant	43	14.4
Daily labor	11	3.7
Private employee	19	6.4
**Monthly income**		
≤1000	6	2.0
1001-3000	91	30.5
3001-5000	76	25.5
5001-7000	40	13.4
>7000	85	28.5

### Obstetric characteristics of respondents

**Figure 1:**
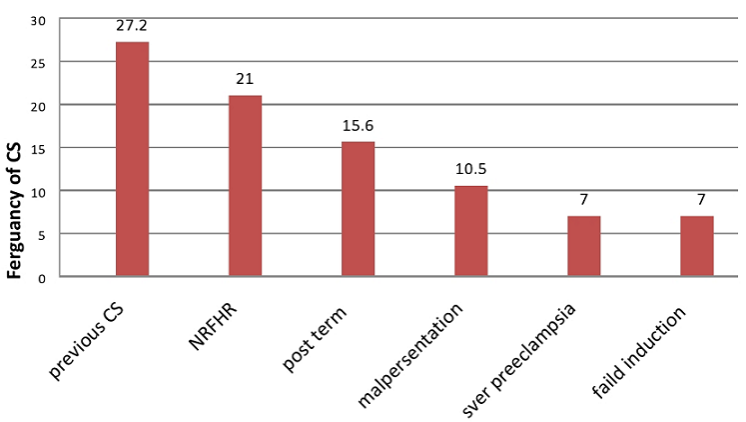
indication of caesarean section among women who gave birth by caesarean section in public and private hospitals, Addis Ababa, Ethiopia 2017

Out of the total respondent more than half 169(56.7%) were gravid II-IV. Majority of respondent 287 (96.3%) had a history of antenatal care (ANC) service utilization for recent delivery. Majority 254(85.2%) of mothers the gestational age were the term, 24(8.1%) preterm and 20(6.7%) were post term. Only 5(3.3%) of participants had previous still birth and 33 (11.1%) of women had history of previous miscarriage. The most common indication for CS in this study was Previous caesarean 31(27.2%), NRFHR 24(21.0%), followed by post term pregnancy 18(15.6%) (Table 2, Figure 1).

**Table 2:** obstetric characteristics of women who gave birth in public and private hospitals, Addis Ababa, Ethiopia 2017 (n=298)

Variables	Frequency	Percentage (%)
**Gravidity**		
1	122	40.9
2-4	169	56.7
≥5	7	2.3
**Number of ANC**		
1	145	48.7
2	133	44.6
3	16	5.4
≥4	4	1.3
**History of abortion**		
Yes	33	11.1
No	265	88.9
**History of Fertility**		
Yes	7	2.3
No	291	97.7
**Gestational age in week**		
<37 week	24	8.1
37-42 week	247	85.2
>42 week	27	6.7
**Mode of delivery**		
vaginal delivery	184	61.7
Caesarian section	114	38.3
**Types of CS**		
Emergency	67	58.8
Elective	47	41.2
**Current status of CS**		
Primary	73	64.0
Repeat	41	36.0
**Number of previous CS**		
1	21	51.2
2	14	34.1
≥ 3	6	14.6
**Fetal weight**		
<2500	59	19.2
2500-3999	234	76.2
≥4000	14	4.5
**Do you have intention to use maternity service after C/S**		
Yes	90	79
No	24	21

### Factors associated with caesarean section

The multivariate logistic regression showed that. Respondent age, educational status of the respondent. Type of health facility and having risk factors were some of the factors significant associated with Caesarean section at P-value of <0.05. Respondent who were collage and above were 3.45 times more likely utilize caesarean section than illiterate [AOR=3.45(95%CI; 1.11.59, 10.77)]. Respondents who were found in age group of 20-29 were 0.47 times more likely associated with caesarean section than others [AOR=0.47 (95%CI; 0.28, 0.79)]. Respondents who were gave birth in private health institution were 1.48 times more likely associated with caesarean section as compared to mothers who were gave birth in governmental health facility [AOR = 1.48 (95%CI; 1.84, 2.59)], respondent who were having risk factors were 2.86 times more likely associated with caesarean section as compared with respondent without risk factors [AOR = 2.86 (95%CI; 1.96, 3.42)] (Table 3).

**Table 3:** factors associated with caesarean section delivery in bivariate and multivariate logistic regression among women who gave birth in public and private hospitals, Addis Ababa, Ethiopia 2017

Variable	Caesarean section		COR (95% CI)	AOR (95% CI)
	Yes	No		
**Age**				
15-19	2	2	0.98(0.183,5.246)	1.028(0.132,8.005)
20-29	62	133	0.457(0.301,0.693)*	0.47(0.28,0.79)*
>29	50	49	1	1
**Educational status**				
No formal education	5	19	1	1
Primary	24	57	1.6(0.639,4.009)	1.749(0.552,5.540)
Secondary	43	64	2.553(1.050,6.205)*	2.813(0.925,8.552)
College & above	42	44	3.627(1.475,8.919)*	3.456(1.109,10.764)*
**Type of facility**				
Government	68	132	1	1
Private	46	52	1.77(1.135,2.597)*	1.48(1.84,2.59)*
**Having risk factors**				
Yes	86	28	3.20(3.51,4.26)*	2.86(1.96,3.42)*
No	28	156	1	1
**Gestational age**				
<37	8	16	0.211(0.64,0.688)*	0.265(0.078,0.899)*
37-42	87	160	0.229(0.096,0.544)*	0.219(0.090,0.535)*
>42	19	8	1	1
**Intention to use maternity service after C/S**				
Yes	56	34	1	1
No	17	7	1.46(0.56-3.84)	2.4(0.72-2.09)

* Statically associated at p<0.05

## Discussion

Caesarean section is a life-saving procedure for both the mother and the baby. Delay in deciding for it may be detrimental for both. On the other hand, the premature and wrong decision may increase the maternal and fetal morbidity and mortality. The purpose of this study was to determine the prevalence of caesarean section and to identify factors leading to CS in Addis Ababa hospitals. The prevalence of women undertaking caesarean section in this study was 38.3%. This finding is consistent with studies conducted in Harare, Eastern part of Ethiopia 34% [20]. This study is also higher than the same study conducted in Yekatit 12 Hospital, Addis Ababa, Ethiopia 5.5% [21]. The discrepancy might be due to a large sample size of the previous study and lack of guide lines and recommendation to the lower high rate of Caesarean section. This finding is also higher than the same study which was conducted in Jimma Hospital; south-western Ethiopia showed that the rate of caesarean section was 8% [22]. The discrepancy might be due to study period difference and this study includes both private and public health facility but the previous study was only public hospitals. This finding is also higher than the same study which was conducted in Tikur Anbessa Teaching hospital, Ethiopia 10% [23]. The discrepancy might be due to study site difference, large sample size of previous study and study design difference. In this study the most common induction for caesarean was pervious caesarian section 27.2%, NRFHR 21%, post term pregnancy 15.6% and malpresentations/malposition 10.5% .This study is consistent with others study which was conducted in another part of Ethiopia [22-24]. According to this study respondents who were found in the age group of 20-29 were more likely utilize caesarean section than others. This finding was different from the same study which was conducted in Felegehiwot referral hospital, Bahir Dar, Ethiopia, showed that respondents who were found in the age group of 15-20 were more likely associated with caesarean section than others [24]. The finding of this study is higher than study conducted in Northwestern Nigeria which show that the prevalence of CS was 11.3% [25]. The discrepancy might be due to socio economic and cultural difference.

## Conclusion

In this study, both obstetric and background Characteristics of mothers were the main reasons that leading to caesarean Section. An increase in the rates of caesarean section delivery is a burden on the health system. Unnecessary caesarean delivery also put a strain on family and may complicate maternal and child health. Therefore, the decision to perform a C-section delivery must be chosen carefully and should not be profit oriented. Early identification of risk age group, clear, compelling and well-supported decision of doctors to perform caesarean section, government intention to develop better health care infrastructure and strict vigil on the private health may help to reduce the high and increasing rate of caesarean delivery.

### What is known about this topic

- Previous study focused only governmental health institution;
- Breast cancer is the leading cause of mortality from all types of cancer occurring among women of reproductive age groups in Ethiopia followed by cervical cancer.

### What this study adds

- This study includes both governmental and private health institutions;
- This study focused both the prevalence and factors associated with caesarian section;
- This study includes referral and teaching hospitals.

## Competing interests

The authors declare no competing interests.
